# Supplementary material for: Mechanisms governing the pioneering and redistribution capabilities of the non-classical pioneer PU.1
Source: Nat Commun. 2020 Jan 21;11:402. doi: 10.1038/s41467-019-13960-2 (PMC6972792; doi:10.1038/s41467-019-13960-2)
Supplement: Supplementary file 7 — Source data [file 41467_2019_13960_MOESM7_ESM.zip › Source_Data/FigureS2/FigureS2B_MotifScanOutput/KmeansCluster2/homerResults/motif30.similar.html]

motif30

## Information for motif30

T
G
C
A
A
G
T
C
C
G
T
A
A
G
T
C
G
A
C
T
A
G
C
T
G
C
A
T
G
A
C
T
C
G
T
A
C
T
G
A
T
C
G
A
G
A
T
C
G
T
A
C
G
C
T
A
  
Reverse Opposite:  

C
A
G
T
C
A
T
G
C
T
A
G
A
G
C
T
A
G
C
T
G
A
C
T
C
T
G
A
C
G
T
A
C
T
G
A
C
T
G
A
T
C
A
G
C
G
A
T
C
T
A
G
A
C
G
T
  

|  |  |
| --- | --- |
| p-value: | 1e-42 |
| log p-value: | -9.754e+01 |
| Information Content per bp: | 1.756 |
| Number of Target Sequences with motif | 92.0 |
| Percentage of Target Sequences with motif | 1.83% |
| Number of Background Sequences with motif | 79.4 |
| Percentage of Background Sequences with motif | 0.20% |
| Average Position of motif in Targets | 107.2 +/- 29.9bp |
| Average Position of motif in Background | 103.2 +/- 59.7bp |
| Strand Bias (log2 ratio + to - strand density) | 0.5 |
| Multiplicity (# of sites on avg that occur together) | 1.00 |
| Motif File: | file (matrix) reverse opposite |

### Similar de novo motifs found

|  |  |  |  |  |  |  |  |
| --- | --- | --- | --- | --- | --- | --- | --- |
| Rank | Match Score | Redundant Motif | P-value | log P-value | % of Targets | % of Background | Motif file |
| 1 | 0.962 | G A T C G C T A A G T C G A C T A G C T G C A T G A C T C G T A C G T A T G C A A T G C G T A C G T C A | 1e-41 | -96.272657 | 1.43% | 0.09% | motif file (matrix) |
| 2 | 0.894 | T C G A G T A C A G C T A C G T C G A T G A C T C G T A G C T A G T C A A T G C G T C A T G C A | 1e-38 | -88.180526 | 1.95% | 0.27% | motif file (matrix) |
| 3 | 0.893 | A G T C C G T A A G T C A C G T C G A T A C G T A G C T C G T A C G T A C T G A A G T C | 1e-30 | -70.178074 | 1.19% | 0.11% | motif file (matrix) |
| 4 | 0.858 | C G T A A G T C A G C T C G A T A C G T A G C T C G T A C G T A C G T A A G T C | 1e-26 | -61.654733 | 1.53% | 0.26% | motif file (matrix) |
| 5 | 0.795 | A G T C A G C T A C G T A C G T A C G T C G T A C G T A C G T A A G T C | 1e-18 | -43.211462 | 1.69% | 0.46% | motif file (matrix) |
